# Effect of Riociguat on Adenine-Induced Chronic Kidney Disease in Rats

**DOI:** 10.3390/biology14020161

**Published:** 2025-02-06

**Authors:** Aly M. Abdelrahman, Raya Al Maskari, Haytham Ali, Priyadarsini Manoj, Yousuf Al Suleimani

**Affiliations:** 1 Department of Pharmacology and Clinical Pharmacy, College of Medicine and Health Sciences, Sultan Qaboos University, P.O. Box 35, Muscat 123, Oman; abdelrahman@squ.edu.om (A.M.A.); r.maskari@squ.edu.om (R.A.M.); priyadarsinimanoj@gmail.com (P.M.); 2 Department of Animal and Veterinary Sciences, College of Agricultural and Marine Sciences, Sultan Qaboos University, P.O. Box 35, Muscat 123, Oman; h.ali@squ.edu.om

**Keywords:** adenine, sGC stimulators, riociguat, chronic kidney disease, blood pressure

## Abstract

Riociguat is a soluble guanylate cyclase (sGC) activator that increases the levels of cyclic guanosine monophosphate (cGMP). cGMP is known to play a key role in regulating kidney function. This research sought to investigate the possible protective effects of riociguat on the kidneys in the context of chronic kidney disease (CKD). CKD was induced in male Wistar rats through adenine administration. A total of 24 rats were allocated into four groups and administered treatments over a period of 35 days. Group 1 received a normal diet and a vehicle (carboxymethylcellulose (0.5%)), serving as the control. Group 2 received adenine (0.25% *w*/*w*) in the feed and a vehicle. Groups 3 and 4 received adenine in the feed (0.25% *w*/*w*) plus riociguat (3 mg/kg/day) and riociguat (10 mg/kg/day), respectively. Adenine administration significantly elevated systolic blood pressure, plasma creatinine, urea, and neutrophil gelatinase-associated lipocalin (NGAL). Furthermore, adenine reduced creatinine clearance and increased the urinary albumin-to-creatinine ratio and urinary N-Acetyl-β-D-Glucosaminidase (NAG). Histopathologically, adenine caused renal tubular necrosis and fibrosis. Furthermore, adenine elevated the plasma concentration of interleukins (IL-1β and IL-6) and tumor necrosis factor-alpha (TNF-α). Adenine significantly increased renal malondialdehyde (MDA) and reduced glutathione reductase (GR), superoxide dismutase (SOD), catalase (CAT), and total antioxidant capacity (TAC). Treatment with riociguat attenuated adenine-induced hypertension, improved kidney function, and ameliorated histopathological changes. Riociguat also reduced kidney injury markers, inflammation, and renal oxidative stress. The renoprotective effect of riociguat is probably due to anti-inflammatory and antioxidant actions. This indicates that riociguat may have the potential to slow the progression of kidney damage in chronic kidney disease (CKD).

## Simple Summary

Chronic kidney disease (CKD) is a serious health condition that affects millions of people, often leading to life-threatening complications. This study investigated whether riociguat, a drug known to improve blood flow and reduce stress on organs, could protect the kidneys from damage caused by CKD. Using a rat model, we induced kidney damage by adding adenine to their diet and then treated the rats with different doses of riociguat. Our findings showed that riociguat helped lower high blood pressure caused by CKD, improved kidney function, and reduced tissue damage in the kidneys. It also decreased the markers of inflammation and oxidative stress, which are major contributors to CKD progression. These results suggest that riociguat could be a valuable add-on treatment for slowing kidney damage in people with CKD, offering hope for better management of this challenging disease.

## 1. Introduction

Chronic kidney disease (CKD) is a complex disease characterized by inflammation, oxidative stress, and progressive fibrosis. Patients with CKD often have elevated levels of inflammatory markers like interleukin-6 (IL-6), tumor necrosis factor-alpha (TNF-α), and C-reactive protein (CRP), which drive disease progression [1]. Oxidative stress, caused by an imbalance between reactive oxygen species (ROS) and the body’s antioxidant defenses, also plays a significant role in kidney damage, contributing to endothelial dysfunction, podocyte injury, and tubular cell death [2]. While there has been significant progress in understanding CKD’s underlying mechanisms, there remains a need for new treatments that can simultaneously address inflammation, oxidative stress, and kidney blood flow abnormalities.

Soluble guanylate cyclase (sGC) is an enzyme that aids in the production of cyclic guanosine monophosphate (cGMP) when it interacts with nitric oxide (NO). NO is a key signaling molecule involved in essential physiological processes like relaxing blood vessels, preventing platelet aggregation, and supporting cell growth [3]. The presence of sGC in various tissues, such as the cardiovascular system, brain, lungs, and kidneys, suggests it could have broader therapeutic uses [4,5]. In the kidneys, sGC is found in structures like glomerular arterioles, granular cells, descending vasa recta, fibroblasts, podocytes, and mesangial cells [5]. The NO/sGC/cGMP pathway is crucial for maintaining kidney function, as it regulates glomerular filtration rate (GFR), renal blood flow, and sodium excretion. When this pathway becomes dysregulated, it can contribute to the development of CKD [6,7].

Riociguat, a medication that stimulates sGC, works through two mechanisms. It enhances the enzyme’s response to NO and can also directly activate sGC without needing NO [8]. In clinical settings, riociguat is primarily used to treat pulmonary hypertension [9]. Preclinical studies indicate that sGC stimulators and activators may have protective effects in different models of kidney injury, including CKD caused by unilateral ureteral obstruction in mice [10], Dahl salt-sensitive rats on high-salt diets [11], hypertensive renin-transgenic rats treated with NG-nitro-L-arginine methyl ester (L-NAME), and rats that have undergone 5/6 nephrectomy [12].

Although the adenine-induced CKD model differs from human CKD in its underlying cause, it replicates many features of the disease, such as tubular atrophy, interstitial fibrosis, and inflammation. This makes it a valuable preclinical model for studying CKD [13]. Researchers have used this model to test the kidney-protective effects of various drugs, demonstrating its utility in exploring how CKD progresses [13,14,15,16,17]. However, few studies have investigated how sGC stimulators like riociguat affect this model, leaving gaps in understanding their impact on kidney inflammation, oxidative stress, and functional decline in CKD.

This study aimed to examine how riociguat affects kidney function, blood pressure, and the markers of inflammation and oxidative stress in the adenine-induced CKD model. By exploring the therapeutic potential of sGC stimulation in this context, this study hopes to provide insights into how riociguat protects the kidneys and its potential role in managing CKD.

## 2. Material and Methods

### 2.1. Chemical Reagents

Adenine was purchased from Sigma (St. Louis, MO, USA), and riociguat was obtained from ZhiShang Chemical, Jinan, China. All other chemicals and reagents used in the study were of the highest commercially available purity.

### 2.2. Animals

Wistar male rats (280–400 g) were provided from the animal house at Sultan Qaboos University (SQU) and were kept in a room with suitable conditions of temperature, humidity, light–dark cycle, food, and water. The experimental procedures received approval from the University Ethical Committee for animal use in research (approval code: SQU/EC-AUR/2021-2022-13) and were conducted following national and international regulations.

### 2.3. Experimental Design

Rats were divided into four groups (*n* = 6 each), and treated as follows.

For Group 1, the rats received the standard diet and were given oral carboxymethylcellulose (0.5%) for 35 days and served as control.

For Group 2, the rats received adenine in their feed at a dose of (0.25% *w*/*w*) and were given oral carboxymethylcellulose (0.5%) for 35 days.

For Groups 3 and 4, the rats were fed adenine (0.25% *w*/*w*) as in Group 2 and additionally received oral riociguat at doses of 3 or 10 mg/kg/day. The riociguat was suspended in 0.5% carboxymethylcellulose and administered for 35 days.

The adenine and riociguat doses were chosen according to previous studies [9,16].

### 2.4. Physiological Measurements

Body weight and blood pressure were recorded at the beginning and end of the treatment. At the end of the treatment, rats were housed in metabolic cages to collect 24 h urine samples. Blood pressure was measured using the tail-cuff method, as described in a previous study [18]. Two weeks prior to the start of the experiment, the rats were familiarized with the blood pressure recording procedure using the Blood Pressure Analysis System™ (BP-2000 SERIES II, Visitech Systems, Apex, NC, USA). During this process, conscious rats were gently placed in a restrainer positioned on a warming pad and given 15 min to acclimate before measurements were taken. The rat’s tail was inserted into a tail cuff, which was inflated and released three times to help the animal adjust to the procedure. Blood pressure readings were recorded at both the beginning and end of the experimental period.

### 2.5. Biochemical Analysis

Blood samples were drawn from the abdominal aorta after the rats were anesthetized with ketamine at a dose of 75 mg/kg and xylazine at 5 mg/kg.

The blood was then centrifuged, and the plasma was stored for further analysis. Plasma urea, creatinine, and uric acid levels, along with urine creatinine and albumin, were measured as described in previous studies [19]. Briefly, the collected blood samples were centrifuged at 4000 rpm for 5 min to separate the serum, which was then used to measure key markers, such as N-acetyl-β-D-glucosaminidase (NAG), tumor necrosis factor-alpha (TNF-α), interleukin-6 (IL-6), and interleukin-1 β (IL-1β), and kidney function indicators, including creatinine, BUN, uric acid, and neutrophil gelatinase-associated lipocalin (NGAL). The kidneys were excised for histopathological evaluation and to assess oxidative stress markers like malondialdehyde (MDA), glutathione reductase (GR), total antioxidant capacity (TAC), superoxide dismutase (SOD), and catalase (CAT). For biochemical assays, kidney tissue was homogenized in a phosphate buffer (pH 7.4) containing a protease inhibitor (1 µg/mL). The homogenate was centrifuged at 800× *g* for 5 min at 4 °C to obtain the supernatant for TAC and MDA analyses. The remaining homogenate was further centrifuged at 10,500× *g* for 15 min at 4 °C to isolate the post-mitochondrial supernatant (PMS), which was used for SOD, GR, and CAT assays.

The plasma levels of IL-1β and NAG were assessed using kits from Cusabio Biotech Co. Ltd. (Wuhan, China). NGAL, IL-6, and TNF-α were measured with kits purchased from Thermo Fisher Scientific, Inc. (Waltham, MA, USA). The animals were euthanized by an overdose of ketamine and xylazine, and the kidneys were collected, blotted dry, and weighed. Portions of the right kidney were fixed in formalin for histopathological analysis, while the remaining kidney tissues were wrapped in aluminum foil, flash-frozen in liquid nitrogen, and stored at −80 °C for biochemical assays. Total TAC and MDA were assessed using kits from MyBioSource, Inc. (San Diego, CA, USA). Renal SOD and GR activities were quantified using colorimetric kits from Biovision (Milpitas, CA, USA), while renal catalase levels were measured with kits from Thermo Fisher Scientific, Inc. (Waltham, MA, USA). Relative kidney weight and creatinine clearance were calculated as previously reported [16]. Creatinine clearance was measured using the urine collection formula:

Urinary creatine (µmol/L) × Urine volume (mL/24 h)/Plasma creatinine (µmol/L) × 1440

### 2.6. Histopathological Analysis 

The kidneys were fixed in 10% neutral buffered formalin, and tissue sections measuring 4 μm in thickness were stained with hematoxylin and eosin (H&E), as well as Picro-Sirius red (ab150681, Abcam, Cambridge, UK). A semi-quantitative scoring method, adapted from [15], was used to evaluate renal tubular necrosis on a scale of 0 to 4, where 0 indicates normal tissue with no necrosis, 1 represents less than 10%, 2 indicates 10–25%, 3 covers 26–75%, and 4 signifies greater than 75% necrosis. Fibrosis was quantified by analyzing the Picro-Sirius red-stained sections following the method described in [19]. Briefly, stained slides were analyzed using an Olympus BX51 microscope with a DP70 camera. For each rat, three random photomicrographs of the renal cortex were captured at 40× magnification and saved as TIFF files. Imaging settings were kept consistent. ImageJ^®^ software, version 1.54, was used to analyze the images, converting them to grayscale and isolating red-stained collagen using a hue histogram filter. The fibrosis index was calculated as the percentage of the area stained with Sirius red relative to the total area of each photomicrograph, providing a quantitative measure of collagen content.

### 2.7. Statistical Analysis

Data were analyzed using GraphPad Prism Version 5.03 for Windows (GraphPad Software Inc., San Diego, CA, USA). The results are presented as means ± SEM. Group differences were evaluated using a one-way analysis of variance (ANOVA) with a post hoc Bonferroni’s test. A *p*-value of less than 0.05 was considered statistically significant.

## 3. Results

### 3.1. Physiological Parameters

**Figure 1:**
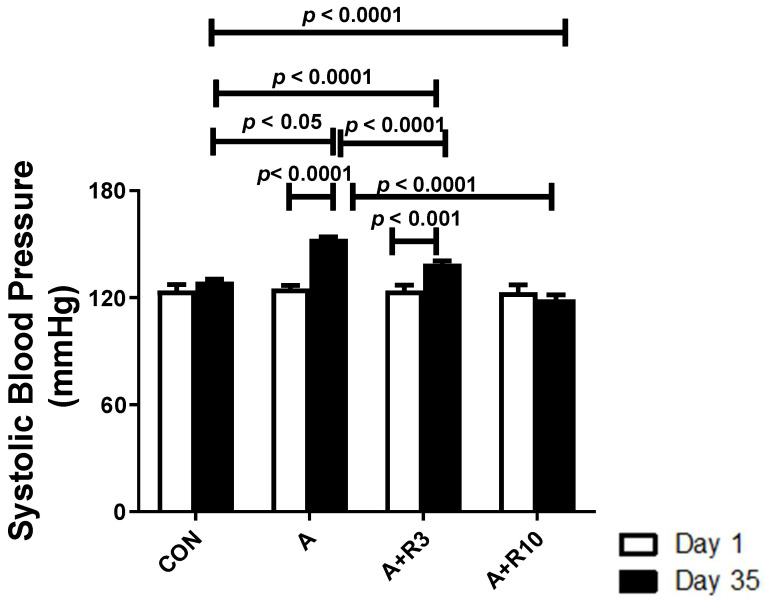
Systolic blood pressure in control rats and rats treated with adenine (A) in the absence and presence of either riociguat (3 mg/kg/day) or (10 mg/kg/day) at the beginning and end of experiment. Data are presented as mean ± SEM (*n* = 6).

Rat body weight decreased and relative kidney weight and urine output were increased by adenine treatment. Treatment with riociguat (3 and 10 mg/kg/day) attenuated the decrease in body weight and the increase in urine output. Riociguat attenuated the increase in relative kidney weight, but this was significant only with riociguat (10 mg/kg/day) (Table 1). There were no significant differences in baseline systolic blood pressure among the groups. The systolic blood pressure was significantly increased in the adenine group (153 ± 1.1 mmHg) compared to the control group (129 ± 1.4 mmHg). Riociguat (3 mg/kg/day) significantly reduced systolic blood pressure (139 ± 1.6 mmHg), but riociguat (10 mg/kg/day) abolished the increase in systolic blood pressure (119 ± 2.6 mmHg) (Figure 1).

### 3.2. Kidney Function, Inflammatory Markers, and Oxidative Stress

**Figure 2:**
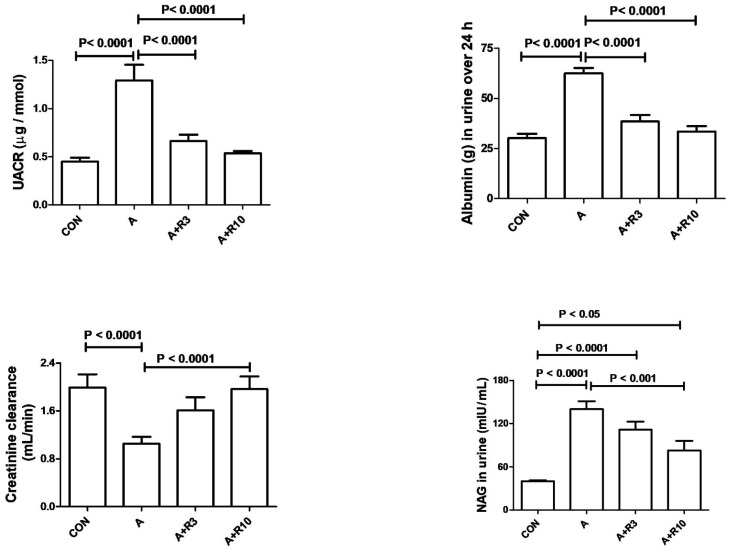
Urinary albumin-to-creatinine ratio (UACR), urinary 24 h albumin, creatinine clearance, and urinary N-acetyl-β-D-glucosaminidase (NAG) in control rats and rats treated with adenine (A) in the absence and presence of either riociguat (3 mg/kg/day) or (10 mg/kg/day) at the beginning and end of experiment. Data are presented as mean ± SEM (*n* = 6).

**Figure 3:**
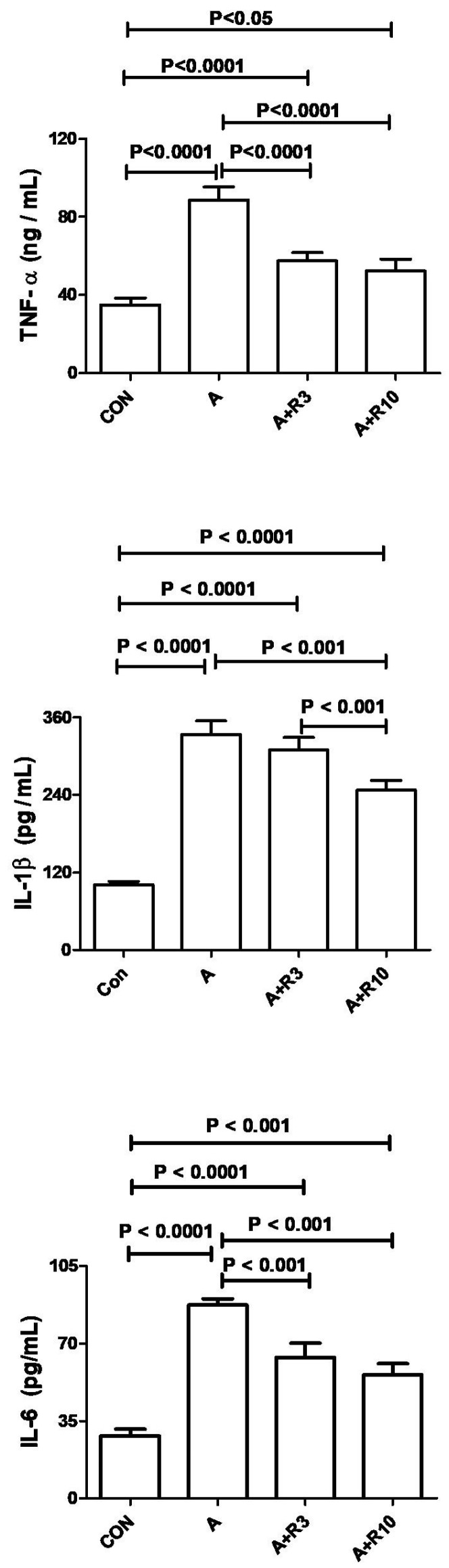
The plasma levels of interleukins (IL-1β and IL-6), along with tumor necrosis factor (TNF-α), were measured in both control rats and those given adenine (A) in the absence and presence of either riociguat (3 mg/kg/day) or (10 mg/kg/day) at the beginning and end of experiment. Data are presented as mean ± SEM (*n* = 6).

**Figure 4:**
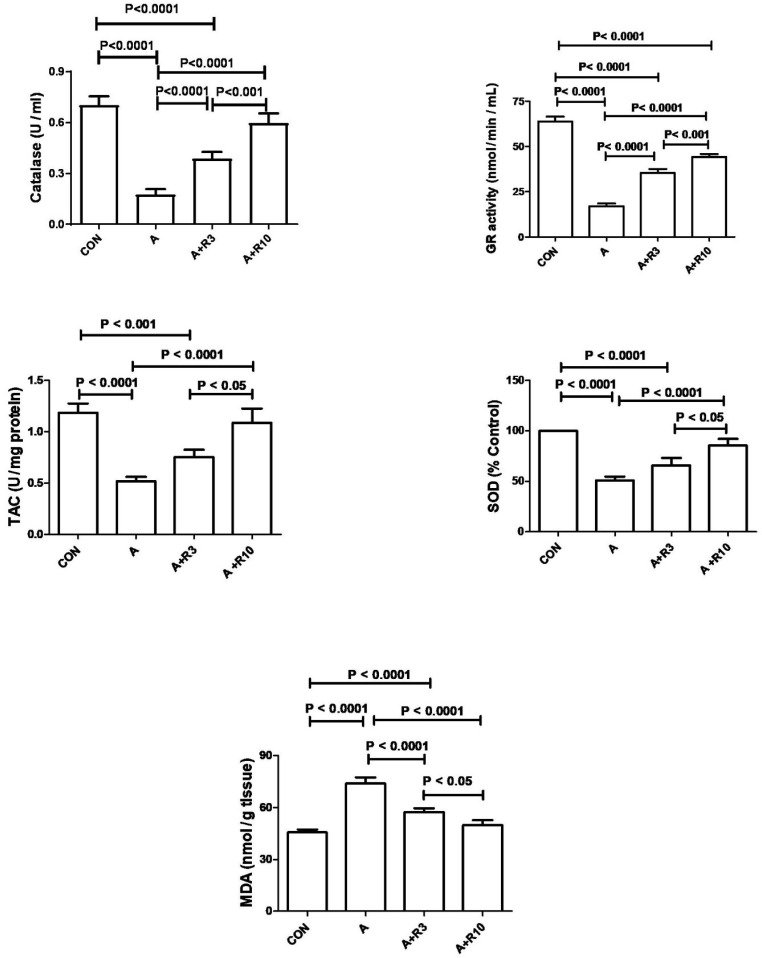
The renal levels or enzymatic activity of SOD, CAT, TAC, GR, and MDA were measured in control rats and those treated with adenine (A) in the absence and presence of either riociguat (3 mg/kg/day) or (10 mg/kg/day) at the beginning and end of experiment. Data are presented as mean ± SEM (*n* = 6). SOD refers to superoxide dismutase, CAT to catalase, TAC to total antioxidant capacity, GR to glutathione reductase, and MDA to malondialdehyde.

Plasma urea, creatinine, uric acid, NGAL, urinary albumin/creatinine ratio, 24 h albumin, and NAG were increased by adenine treatment, while creatinine clearance was reduced (Table 2; Figure 2). Figure 3 shows that adenine increased plasma IL-6, IL-1β, and TNF-α. Figure 4 shows that adenine increased oxidative stress, as it significantly decreased renal catalase, GR, SOD, and TAC and increased renal MDA levels. Riociguat, in a dose-dependent pattern, significantly reduced adenine-induced changes in kidney function, kidney injury markers, inflammatory markers, and oxidative stress. However, the increase in creatinine clearance, decrease in urinary NAG and plasma IL-1β, and increase in renal TAC and SOD were not statistically significant with riociguat (3 mg/kg/day) (Table 2, Figure 2, Figure 3 and Figure 4).

### 3.3. Histopathology

**Figure 5:**
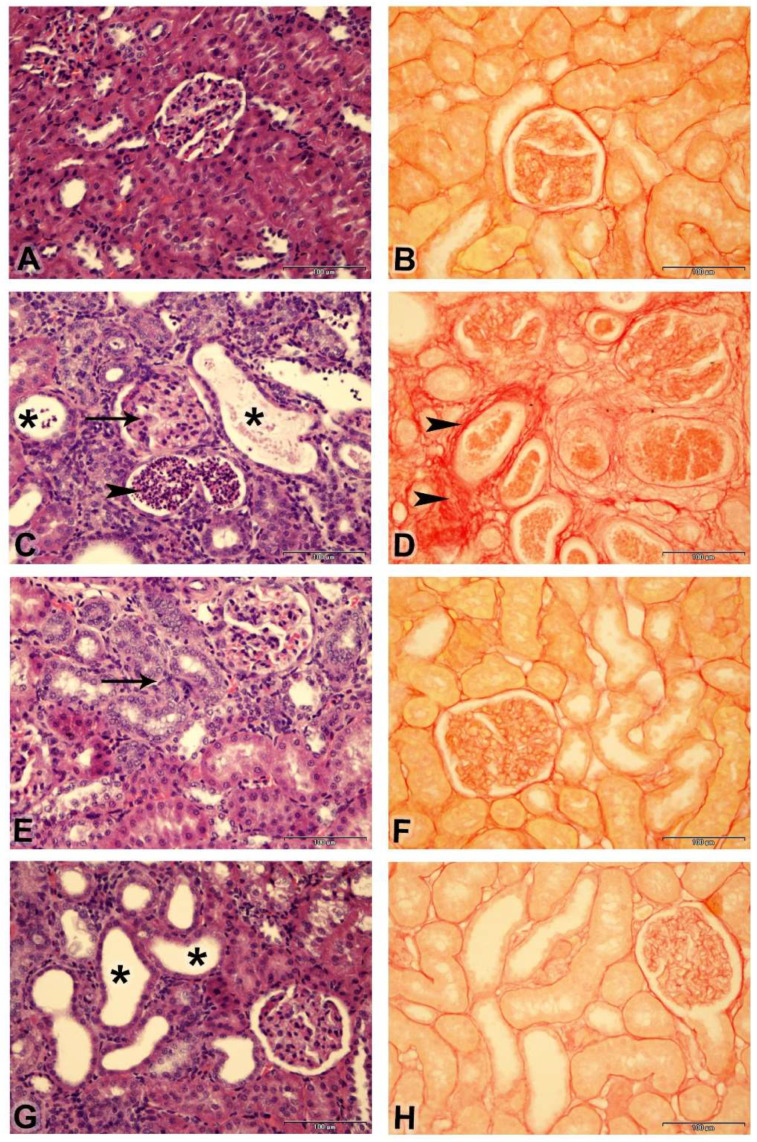
Photomicrographs of kidneys (Bar = 100 µm, (**A**,**C**,**E**,**G**) stained with: H&E; (**B**,**D**,**F**,**H**): stained with Picro-Sirius red): (**A**) Control group showing normal renal histology (lesion score “0”). (**C**) Adenine group showing shrunken glomeruli (arrow), tubular cystic dilatation (asterisk), basophilia of the renal tubules, and cellular casts (arrowheads) (lesion score 4); (**E**) Adenine + riociguat (3 mg/kg/day) showing marked basophilia of renal tubules (arrow) (lesion score “2”); (**G**) Adenine + riociguat (10 mg/kg/day) showing dilatation of few renal tubules (asterisk) (lesion score 3). ((**B**), control group); ((**D**), adenine group); ((**F**), adenine + R3) ((**H**), adenine + R10) showing collagen fibers in red (arrowheads) and non-collagen structures in yellow.

A histopathological examination of the renal tissues and semiquantitative analysis of renal tubular necrosis are shown (Figure 5A,C,E,G and Table 3). Adenine treatment induced histopathological lesions with an increased tubular necrosis lesion score of “4” in comparison to “0” in the control group. Riociguat treatment improved renal histopathology, as demonstrated by lesion scores of “2” and “3” in the third and fourth groups, respectively.

Collagen fibers, in red, and non-collagen structures, in yellow, were visualized using Picro-Sirius red stain, as presented in Figure 5B,D,F,H. The degree of fibrosis was elevated in the adenine group and decreased with riociguat treatment (Table 3).

## 4. Discussion

CKD affects more than 10% of the population in the world, with more than 800 million patients [20], and new therapeutic options are required. Diwan et al. [17] reviewed the available rat models of CKD that included an adenine-induced model. Yang et al., [13] reviewed the advantages and disadvantages of the adenine-induced model and suggested that its advantages include that it is simple to administer in the feed or by gavage, surgery is not required as in other models, so mortality is less, and it can produce a stable disease course. Since disturbances in the NO/sGC/cGMP pathway can lead to kidney dysfunction [6], sGC stimulators and activators can be a potential therapy for CKD [21]. sGC stimulators and sGC activators are two different classes of agents that can activate sGC in a NO-independent manner. They act on the sGC/cGMP pathway by binding to the heme-containing sGC and the heme-free sGC, respectively [22]. By its dual action on sGC, riociguat causes dilatation of the blood vessels and has anti-proliferative and anti-fibrotic effects [11]. In the present study, adenine increased systolic blood pressure, which was reported by others [18,23,24,25,26]. Riociguat dose-dependently reduced systolic blood pressure. The low dose of riociguat significantly reduced systolic blood pressure, but the increase in systolic blood pressure in the rats treated with the riociguat (10 mg/kg/day) was completely abolished. This is in accordance with a previous result that showed that riociguat (3 mg/kg/day) reduced systolic blood pressure in hypertensive renin-transgenic rats treated with L-NAME, while riociguat (10 mg/kg/day) prevented the increase in blood pressure [12]. This is due to riociguat being a stimulator of sGC, leading to increased cGMP [3]. cGMP has an important role in the regulation of vascular tone [8] and causes relaxation of vascular smooth muscle [10]. Riociguat was shown to cause vasodilatation of the saphenous artery ring of rabbits [10]. In addition, riociguat injection caused vasodilatation and hypotension in pigs [3]. Mittendorf et al., [9] showed that riociguat caused relaxation of a precontracted rabbit aorta and reduced blood pressure in spontaneously hypertensive rats. Vericiguat, an sGC stimulator, caused vasodilatation of the saphenous artery and aortic rings of rabbits and attenuation of hypertension in blood pressure in L-NAME-treated renin-transgenic rats [27]. This study shows that adenine caused kidney dysfunction as shown by increased plasma creatinine and urea and decreased creatinine clearance. Adenine also increased urinary albumin/creatinine ratio, 24 h albumin excretion, and kidney injury markers, such as plasma NGAL and urinary NAG. Adenine treatment was also shown to increase inflammatory markers and oxidative stress. This is in agreement with previous reports [15,16,18,24]. Increased oxidative stress was shown to be involved in the progression of renal tubulointerstitial injury in this model of CKD [28]. Oxidative stress was also shown to impair the NO/sGC/cGMP pathway in CKD [29]. Adenine-induced chronic kidney disease may be due to the metabolism of adenine to 2,8-dihydroxyadenine, which accumulates crystal deposits in the renal tubules and induces renal tubular injury, inflammation, and oxidative stress [13]. Histopathological examination of renal tissues in the present study revealed that adenine treatment was associated with increased renal tubular necrosis and fibrosis, indicating tubular injury. This is in agreement with previous studies showing that this model of CKD is associated with tubular injury, as indicated by increased dilated tubules, tubular lumen cellular debris, and fibrosis [13]. The increase in urinary NAG by adenine treatment confirms that the renal tubules are injured in this model of CKD, since urinary NAG is increased mainly due to injury to the proximal tubule epithelial cells [30]. The present study showed that riociguat, in a dose-dependent manner, attenuated the changes induced by adenine on kidney function, albuminuria, and markers of kidney injury. Riociguat (10 mg/kg/day), but not riociguat (3 mg/kg/day), significantly increased creatinine clearance. In addition, riociguat attenuated an adenine-induced increase in the level of proinflammatory markers such as TNF-α, IL-1β, and IL-6. Riociguat also attenuated adenine-induced oxidative stress, as shown by increases in renal catalase, SOD, TAC, and GR and a decrease in renal MDA. Riociguat also attenuated the tubular necrosis lesion score and reduced renal fibrosis. However, riociguat (10 mg/kg/day) did not confer further attenuation in the histopathological lesions than riociguat (3 mg/kg/day). The reason for the discrepancy between these histopathology results and the dose-dependent effect of riociguat on kidney function is not clear. The reduction in blood pressure and the renoprotective effect of riociguat are in accordance with previous studies that showed that riociguat (1, 3, and 10 mg/kg/day for 14 days) ameliorated kidney injury and fibrosis, reduced oxidative stress and proinflammatory markers in unilateral ureteral obstruction induced-CKD in mice. However, contrary to our study, improvement of the kidney injury scale was more pronounced with the higher dose [10]. Riociguat (3 and 10 mg/kg/day for 14 weeks) reduced systolic blood pressure and attenuated glomerulosclerosis and renal fibrosis in Dahl/ss rats maintained on a high-salt diet [11]. Sharkovska et al., [12] showed that riociguat (3 and 10 mg/kg/day) for 18 days improved kidney function and attenuated renal interstitial fibrosis in hypertensive renin-transgenic rats treated with L-NAME, and they also showed that riociguat (15 mg/kg/day) for 15 weeks reduced blood pressure and improved kidney function and attenuated renal fibrosis in rats with 5/6 nephrectomy. In diabetic eNOS knockout mice, riociguat (3 mg/kg/day for 11 weeks) reduced systolic blood pressure and attenuated renal interstitial fibrosis [31]. In addition, Bay 41-2272 increased glomerular cGMP levels, causing the inhibition of mesangial proliferation, glomerular matrix accumulation, and proteinuria in a mesangial proliferative glomerulonephritis model in rats [6]. Vericiguat, a sGC stimulator, showed a renoprotective effect in Dahl/ss fed a high-salt diet [32]. Olinciguat, another sGC stimulator, ameliorated kidney injury in a mouse model of systemic inflammation with sickle cell disease [33]. Cinaciguat, an sGC activator, improved the glomerular filtration rate and attenuated renal fibrosis in streptozotocin-induced Type 1 diabetic mice with and without endothelial nitric oxide synthase (eNOS) knockout [7]. In addition, cinaciguat ameliorated glomerular damage in type 1 diabetic rats [34]. In hypertensive, diabetic, and metabolic rat models of chronic kidney disease, runcaciguat, another sGC activator, reduced proteinuria, markers of kidney damage, and attenuated renal histopathological changes [21]. In a placebo-controlled trial, avenciguat, an sGC activator, led to improvements in albuminuria in CKD patients [35]. In another clinical trial, runcaciguat, an sGC stimulator, improved albuminuria in patients with CKD [36].

The limitations of this study include that the blood and renal levels of NO and GMP were not measured in the different groups to determine the effect of adenine on the NO/cGMP pathway and to confirm the mechanism of the protective effect of riociguat in this model of CKD.

## 5. Conclusions

This study showed that riociguat had a renoprotective effect in adenine-induced CKD. Riociguat caused significant attenuation of the adenine-induced increase in blood pressure, improved kidney function, reduced inflammatory markers and kidney oxidative stress, and ameliorated renal histopathology. This is possibly due to its anti-inflammatory and antioxidant effects. Future work is required to examine the effect of riociguat in different animal models of acute and chronic kidney diseases. In addition, the exact mechanism of this renoprotective effect of riociguat needs to be examined.

## Figures and Tables

**Table 1:** Effect of riociguat (3 mg/kg/day, R3) and (10 mg/kg/day, R10) treatment on some physiological parameters in rats with adenine (A)-induced chronic kidney disease.

Parameters/Treatment	Control	A	A + R3	A + R10
Baseline body weight (g)	350 ± 15.19	350 ± 14.26	350 ± 16.57	350 ± 17.40
Final body weight (g)	369.7 ± 16.4	346.2 ± 14.7	360 ± 19.2	361.8 ± 16.3
Body weight change (%)	5.55 ± 0.81	−0.98 ± 0.42 ^a^	2.68 ± 1.22 ^b^	3.43 ± 0.81 ^b^
Total kidney weight (g)	1.81 ± 0.08	2.58 ± 0.17 ^a^	2.18 ± 0.2	2.03 ± 0.09
Relative kidney weight (%)	0.49 ± 0.01	0.75 ± 0.06 ^a^	0.60 ± 0.03	0.57 ± 0.04 ^b^
Water intake (mL/24 h)	20.2 ± 1.96	39.2 ± 1.74 ^a^	26.5 ± 0.85 ^b^	22.3 ± 1.8 ^b^
Urine output (mL/24 h)	10.3 ± 0.96	31.2 ± 1.22 ^a^	19.0 ± 1.15 ^a,b^	17.7 ± 1.58 ^a,b^

The values presented in the table are expressed as means ± SEM. (*n* = 6). ^a^ denotes significance of control group vs. different groups. ^b^ denotes significance of A alone group vs. A treated other group.

**Table 2:** Select plasma parameters affected by riociguat treatment at doses of 3 mg/kg/day (R3) and 10 mg/kg/day (R10) in rats with chronic kidney disease induced by adenine (A).

Parameters/Treatment	Control	A	A + R3	A + R10
Urea (mmol/L)	4.33 ± 0.21	11.00 ± 0.60 ^a^	7.09 ± 0.53 ^a,b^	5.21 ± 0.19 ^b^
Creatinine (μmol/L)	24.4 ± 0.98	34.3 ± 1.38 ^a^	26.5 ± 1.21 ^b^	22.4 ± 0.37 ^b^
Uric acid (μmol/L)	32.4 ± 1.17	71.8 ± 5.75 ^a^	60.5 ± 3.5 ^a,b^	46.8 ± 1.54 ^a,b^
NGAL (ng/mL)	34.32 ± 3.26	83.73 ± 5.18 ^a^	60.4 ± 5.04 ^a,b^	54.1 ± 4.8 ^a,b^

Data presented as means ± SEM (*n* = 6). ^a^ denotes significance of control group vs. different groups. ^b^ denotes significance of A alone group vs. A treated other group.

**Table 3:** Histopathological changes in kidney sections of rats with adenine-induced chronic kidney injury following riociguat treatment at 3 mg/kg (R3) and 10 mg/kg (R10).

Groups/Finding	Lesion Score (Tubular Necrosis)	Fibrosis Index %
Control	0	5.2
Adenine	4	27.07
Adenine + R3	2	14.16
Adenine + R10	3	15.77

## Data Availability

Data will be made available on request.
